# Retraction: Long noncoding RNA FOXD2-AS1 promotes the malignancy of cervical cancer by sponging microRNA-760 and upregulating hepatoma-derived growth factor

**DOI:** 10.3389/fphar.2022.1129216

**Published:** 2023-01-05

**Authors:** Frontiers Editorial Office

Following publication, concerns were raised regarding the validity of the data in the article. The authors failed to provide the raw data or a satisfactory explanation during the investigation, which was conducted in accordance with Frontiers’ policies. Given the concerns, and the lack of raw data, the editors no longer have confidence in the findings presented in the article.

This retraction was approved by the Chief Editors of Frontiers in Pharmacology and the Chief Executive Editor of Frontiers. The authors have agreed to the retraction.

